# The Medicinal Values of Culinary-Medicinal Royal Sun Mushroom (*Agaricus blazei Murrill*)

**DOI:** 10.1155/2013/842619

**Published:** 2013-10-31

**Authors:** Hang Wang, Zhiming Fu, Chunchao Han

**Affiliations:** School of Pharmacy, Shandong University of Traditional Chinese Medicine, Jinan 250355, China

## Abstract

*Agaricus blazei* Murrill (ABM), a mushroom native to Brazil, is a basidiomycete brown fungus, which is popularly known as “*Cogumelo do Sol*” in Brazil or “*Himematsutake*” in Japan, and there has been a prominent increase in the use of ABM for therapeutic and medicinal purposes. ABM is useful against a variety of diseases like cancer, tumor, chronic hepatitis, diabetes, atherosclerosis, hypercholesterolemia, and so on. In this review, we demonstrated various pharmacological effects of ABM, so that we can use different effects of ABM against different diseases and provide reference for the study of ABM in the future.

## 1. Introduction

Mushrooms have been used in humans' food since ancient times, which are low in calories and high in minerals, vitamins, fibers, and essential amino acids. In recent years, there has been a significant increase in the consumption of mushroom due to an increasing number of studies identifying the therapeutic properties of the substances isolated from various species of these fungi [1].

*Agaricus blazei* Murrill (or *A. brasiliensis*), a mushroom of Brazilian origin, is widely used for nonprescript, medicinal purposes, both as an edible mushroom and in the form of extracts, which has been used as a health care product for the prevention of a wide range of illnesses including cancer, tumor, chronic hepatitis, diabetes, atherosclerosis, and hypercholesterolemia (Table 1). With the development of scientific researches, more and more scientific research have studied the chemical constituents and pharmacological effects of *A. brasiliensis*. The major chemical compounds include polysaccharide, protein, lectins, amino acid, vitamin, and sterols. 

After the *in vivo* and *in vitro* studies, the pharmacological effects of *A. brasiliensis* including antitumor, antiviral, anti-inflammatory, liver protection, antidiabetic, antihyperlipidemic, antiatherosclerosis, antiallergic, and immunomodulating were found. Although the clinical research about pharmacological effects of *A. brasiliensis* is less, *A. brasiliensis* as a complementary and alternative medicine is widely used. 

## 2. Medicinal Values of *A. brasiliensis*

### 2.1. Anticancer Activity

*Agaricus brasiliensis*, a medicinal edible fungus, is widely studied and used because of its significant anticancer activity. In Japan, researchers demonstrated anticancer and immunostimulant effects of *A. brasiliensis* extracts experimentally, and due to the improving consumption of this mushroom in recent years, a considerable effort investigated the putative effects with interesting but still insufficient clinical studies. Some reports showed that polysaccharide is the main component of *A. brasiliensis* for antitumor [2–4].

#### 2.1.1. Studies in Animals

Polysaccharide antitumoral activity has been evaluated most often against allogenic sarcoma 180 in CD-I mice [5]. *A. brasiliensis* (Himematsutake) has stronger antitumor activity against Sarcoma 180 in mice than do polysaccharides from *Ganoderma lucidum*, *Lentinus edodes*, and *Coriolus versicolor* [6]. Mizuno et al. [6] studied the against antitumor polysaccharide Sarcoma 180 from the mycelium of liquid-cultured *A. brasiliensis*. But the isolated polysaccharide did not react with antibodies of antitumor polysaccharides such as lentinan, gliforan, and FIII-2-b, which is one of the antitumor polysaccharides from *A. brasiliensis*. Moreover, the analyses of ^13^C-NMR and GC-MS suggested that this polysaccharide was preliminarily glucomannan with a main chain of **β**-l,2-1inked D-mannopyranosyl residues and **β**-D-glucopyranosyl-3-O-13-D-glucopyranosyl residues as a side chain [7].

However, some reports indicated that agaritine and its derivatives exerted antitumor activity against leukemic cells, mainly U937 cells [8, 9]. Agaritine was fractionated by HPLC from a hot water extract of ABM powder, and the structure was determined by NMR and MS analyses. This compound inhibited the proliferation of leukemic cell lines, especially suppressing the viability of U937 cells* in vitro *[8]. Therefore, the antitumor substances of ABM remain to be further researched. 

#### 2.1.2. Clinical Studies

It has been reported that 100,000–300,000 kg of the dried body of *A. blazei* is produced every year in Japan, and about 300,000–500,000 persons assume the 3–5 g three times a day by a typical hot water extract of *A. blazei* as an adjuvant with cancer chemotherapy drugs for the prevention or treatment of cancer.

### 2.2. Antiviral Activity

The pharmacological effects of *Agaricus brasiliensis* have been mainly related to the presence of polysaccharides and protein polysaccharide complexes [11]. Faccin et al. [12] reported that the extracts of fruiting bodies of ABM, including aqueous and alcohol extracts, and an isolated against polysaccharide from this species displayed antiviral activity poliovirus type 1. de Sousa Cardozo et al. [13] reported the chemical modification of a polysaccharide extracted from* A. brasiliensis *mycelia to obtain its sulfated derivative (MI-S), which presented a promising inhibitory activity against HSV-1 (KOS and 29R (acyclovir-resistant) strains) and HSV-2 strain 333. Furthermore, the sulfated polysaccharide also presented synergistic antiviral effect with acyclovir.

### 2.3. Liver Protection

Hepatic fibrosis is caused by chronic damage to the liver in union with the progressive accumulation of fibrillar extracellular matrix proteins [32–34]. The main causes of hepatic fibrosis in humans include infection by hepatitis B and C, alcohol abuse, and nonalcohol steatohepatitis, and liver cirrhosis can be induced by carbon tetrachloride (CCL_4_) [35, 36]. A few studies have researched that *A. brasiliensis* extract could ameliorate or abrogate CCL_4_-induced liver injury in rats [14, 15].

#### 2.3.1. Studies in Animals

Chen et al. [16] have demonstrated that *A. brasiliensis* extract might serve as an adjuvant in improving the efficacy of hepatitis B vaccines *in vivo. *The results showed that not only a significant increase in the HBcAg-specific antibody response was observed, but also T cell proliferation was observed in mice which received HBcAg DNA vaccine plus *A. brasiliensis* extract [16].

#### 2.3.2. Clinical Studies

Hsu et al. [17] performed a 1-year open-label pilot study to observe whether *A. brasiliensis* extract improves liver function in patients with hepatitis B. They gave the four enrolled patients *A. brasiliensis* extract of 1500 mg daily for 12 months and measured the level of aspartate aminotransferase (AST) and alanine aminotransferase (ALT). At the end of the study, the mean level of AST and ALT decreased from 246.0 (± standard deviation (SD) 138.9) to 61.3 (± SD 32.6) IU/L and 151.0 (± SD 86.9) to 46.1 (± SD 22.5) IU/L, respectively [17]. Although the result of this study showed that *A. brasiliensis* extract can normalize liver function of the 4 patients, this study is just a small sample research.

In addition, Grinde et al. [18] reported the effect on gene expression in peripheral blood cells from four chronic hepatitis C patients, using global (29 k) oligo-based, single channel microarrays. After dates being analyzed, the results suggested that the **β**-glucan part of the *A. brasiliensis* extract was not transported into the blood in appreciable quantities. And although the average (*n* = 5) titre of virus was slightly lower after one week on *A. brasiliensis*, the difference was clearly not significant [18]. So, this result of the study showed that **β**-glucan of the *A. brasiliensis* extract cannot treat HCV. However, one study evaluated the clinical effects and safety on 20 volunteers (50% of men) with elevated **γ**-GTP activity of *A. blazei* condensed liquid (*Agaricus* mushroom extract, ABCL) in the treatment of C-hepatitis. Decreasing effect for serum **γ**-GTP activity was found in 80% of the patients in both sexes after these patients received the ABCL orally, twice a day, for 8 weeks, without any toxicological findings and other side effects [19]. Additionally, Mukai et al. [20] reported three cases of patients with advanced cancer who showed severe hepatic damage, and two of whom died of fulminant hepatitis after taking *A. brasiliensis *extract. Reporters demonstrated that a strong causal relationship between the *A. brasiliensis* extract and liver damage was suggested and, at least, taking the *A. brasiliensis* extract made the clinical decision-making process much more complicated, although several other factors cannot be completely ruled out as the causes of liver damage [20].

### 2.4. Immunomodulating Effect

**Figure 1:**
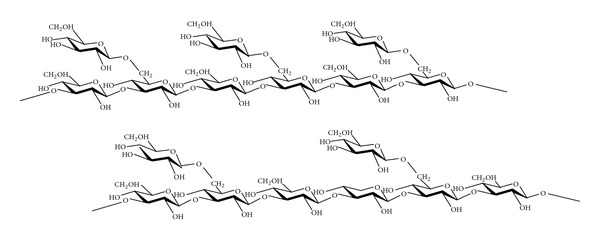
*β*-(1,3-1,6)-D-Glucan.

Polysaccharide is an immunologic adjuvant, which not only can activate the activity of T cells, B cells, NK cells, and other immune cells but also can promote the synthesis of IL-1, IL-2, TNF-**α**, IFN-**γ*,* and NO, regulating the formation of body's antibodies and complement. *A. brasiliensis* contains compounds such as (1→3), (1→6)-**β**-glucans (Figure 1), (1→3)-**α**-glucans, and protein-polysaccharide complexes, which can enhance *in vivo* and *in vitro* cell-mediated immune responses and act as biological response modifiers [21, 22].

#### 2.4.1. Studies in Animals

Lin et al. [23] have established leukemia mice through the injection of WEHI-3 cells and chronically treated mice with *A. brasiliensis*. In their study, results showed that *A. brasiliensis* can promote immune responses in leukemia mice *in vivo* and also can promote T cell proliferation. Furthermore, the* A. brasiliensis* extract significantly enhanced both NK cell activities and phagocytosis of macrophages [23].

#### 2.4.2. Clinical Studies

However, there was a randomized clinical trial on elderly women to ascertain the effects of AbM intake on serum levels of IL-6, IFN-**γ**, and TNF-**α** in community-living seniors [24]. After the study period, no changes from baseline were detectable for any parameter in either group, receiving placebo or AbM dry extract with 900 mg/day for 60 days. Therefore, it showed that AbM extract had no modulating effect on IL-6, IFN-c, or TNF-a levels in elderly females [24].

### 2.5. Antidiabetic Effect

*Agaricus brasiliensis* is rich in polysaccharides and protein, especially **β**-glucans. Kim et al. [25] demonstrated that **β**-glucans and their enzymatically hydrolyzed oligosaccharides (AO) from *A. brasiliensis* show the activities of antihyperglycemic, antihypertriglyceridemic, antihypercholesterolemic, and antiarteriosclerotic indicating antidiabetic activity as a whole in diabetic rats. In this study, diabetic rats were divided into four groups, including normal control, diabetic control, treated group I (**β**-glucans), and treated group II (AO), to contrast the different changes of their body weights. The data suggested that both **β**-glucans and AO might promote insulin secretion from islets as well as the viability and proliferation of islets in diabetic or normal rats. 

In addition, several studies demonstrated that *A. brasiliensis* had an effect on streptozotocin-induced diabetic rats [26–28]. Oxidative stress induced by hyperglycemia possibly causes the dysfunction of pancreatic **β**-cells and various forms of tissue damage in patients with diabetes mellitus. Niwa et al. [28] researched the antidiabetic efficacy and hypoglycemic mechanisms of *Ipomoea batatas* and *A. brasiliensis* in streptozotocin-induced diabetic rats. The results of the study suggested hypoglycemic effects of *Ipomoea batatas* or *A. brasiliensis* due to their suppression of oxidative stress and proinflammatory cytokine production followed by improvement of pancreatic **β**-cells mass [28].

### 2.6. Antileishmanial Effect

**Figure 2:**
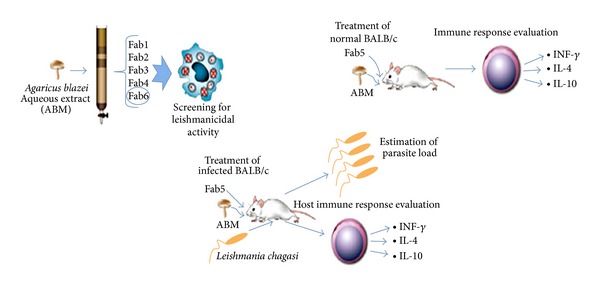
Reference schematic diagram of Valadares et al. using the five fractions obtained from ABM water extract to treat BALB/c mice infected with* Leishmania chagasi.*

Leishmaniasis is a flock of vector-transmitted diseases that are endemic in many tropical and subtropical countries. The current treatment for leishmaniasis has certain side effects, and some drugs are of high cost for the majority of patients. In recent years, *A. brasiliensis* was demonstrated to have antileishmanial activity, and thereinto an *in vitro* antileishmanial activity against *L. amazonens is*,* L. chagasi*, and* L. major* was demonstrated for an *A. blazei* water extract [29]. Valadares et al. [30] studied the therapeutic efficacy induced by the oral administration of *A. brasiliensis* against *Leishmania amazonensis*. The results showed that mice treated with the *A. brasiliensis* presented a 60% reduction in the inflammation of infected footpads as compared to untreated control-infected mice. These treated animals produced significantly higher levels of interferon gamma (IFN-**γ**) and nitric oxide (NO), higher levels of parasite-specific IgG2a isotype antibodies, and lower levels of IL-4 and IL-10 in the spleen and lymph node cell cultures than did the controls [30]. In addition, Valadares et al. [31] used five fractions obtained from *A. brasiliensis* water extract to treat BALB/c mice infected with *Leishmania chagasi in vivo* (Figure 2). The results suggested that the use of Fab5 (molecules >100,000 Da) or *A. brasiliensis*, as compared to control groups, resulted in significant reduced parasite burdens in the liver, spleen, and draining lymph nodes of the infected animals. 

## 3. Other Effects

There are not only these pharmacological effects of *A. brasiliensis*, which are described previously, but also other beneficial effects. The chloroform-soluble extract of *A. brasiliensis* inhibited IL-6 production in PMA plus A23187-induced BMMCs (bone marrow-derived mast cells) to express the anti-inflammatory and antiallergic effects [37]. One study, the first *in vivo* study, showed that *A. brasiliensis* can enhance local and systemic inflammation, upregulate proinflammatory molecules, and enhance leukocyte homing to atherosclerosis sites without affecting the lipoprotein profile [38]. The polysaccharides of *A. brasiliensis* have antitumor, antiviral, and immunomodulating effects as described above. Besides, one study indicated that *A. brasiliensis* polysaccharides could be useful in promoting burn wound healing [39].

There is a clinical study stating that Administration of **γ**-aminobutyric acid (GABA) enriched *A. blazei* (AG-GABA) to mild hypertensive human subjects showed that both systolic and diastolic blood pressure values decreased to statistically significant levels [40]. Maybe there are also many other effects of *A. brasiliensis*, which are not known so far. Therefore, it needs to be further researched.

## 4. Conclusion 

**Figure 3:**
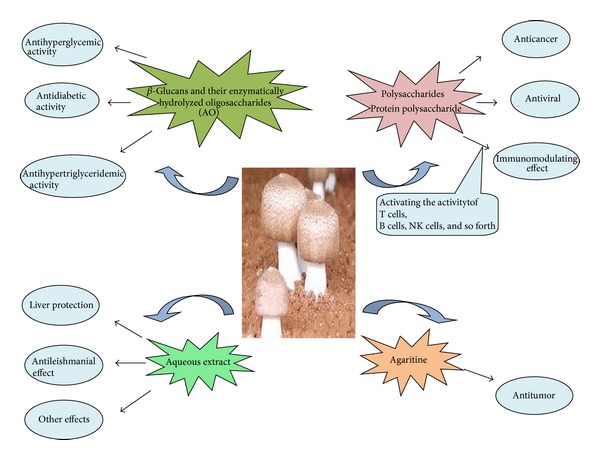
Medicinal values and active compounds of *A. brasiliensis.*

*Agaricus blazei Murrill,* a mushroom of biomedical importance, contains a number of bioactive components, many of them biological are response modifiers which activate our immune systems for a multitude of defensive functions (Figure 3). Polysaccharides of *A. brasiliensis* have been known to have anticancer, antiviral, and immunomodulatory effects, and other substances are probably involved as well. Moreover, **β**-glucans and their enzymatically hydrolyzed oligosaccharides (AO) from *A. brasiliensis* show antihyperglycemic, antihypertriglyceridemic, antihypercholesterolemic, and antiarteriosclerotic activities [25]. Therefore, the pharmacological effects and health function of *A. brasiliensis* are more and more focused on in the world. Although there seems to be clear evidences that ABM extract are rich in **β**-glucans, which presumably contribute to the observed pharmacological activities, isolation, and dose response studies, as well as chemical identification and quantification of specific compounds responsible for the potential benefit from ABM, mushroom ingestion should be fully developed. Careful clinical studies comparing the activity of the whole mushroom extracts, isolated compounds, and epidemiological data still need to determine whether *A. brasiliensis* provides real clinical benefits.

## Figures and Tables

**Table 1:** Medicinal values and active compounds of *A.brasiliensis. *

Medicinal value	Active compounds	References
Anticancer activity	Polysaccharides agaritine	[2–10]
Antiviral activity	Polysaccharides Protein polysaccharide	[11–13]
Liver protection	Aqueous extract	[14–20]
Immunomodulating effect	Polysaccharides	[21–24]
Antidiabetic effect	*β*-glucans and their enzymatically hydrolyzed oligosaccharides (AO)	[25–28]
Antileishmaniasis effect	Aqueous extract	[29–31]
